# BETTER TOGETHER? PERCEIVED PROSPECTIVE MEMORY FAILURES RELATE TO COGNITIVE COLLABORATION IN MIDDLE AND LATE LIFE

**DOI:** 10.1093/geroni/igae098.4131

**Published:** 2024-12-31

**Authors:** Anisha Sharma, Celinda Reese-Melancon, Jennifer Margrett, Riley Wyatt, Erin Harrington, Jennifer Byrd-Craven, Rachael Turner, Peter Rendell

**Affiliations:** Oklahoma State University, Stillwater, Oklahoma, United States; Oklahoma State University, Stillwater, Oklahoma, United States; Iowa State University, Ames, Iowa, United States; Oklahoma State University, Stillwater, Oklahoma, United States; University of Wyoming, Laramie, Wyoming, United States; Oklahoma State University, Stillwater, Oklahoma, United States; University of Nebraska Kearney, Kearney, Nebraska, United States; Australian Catholic University, Australian Catholic University, Victoria, Australia

## Abstract

Research examining everyday memory among older couples indicates that partners rely on one another for cognitive support (e.g., Harris et al., 2022). We built upon this body of work to determine whether perceptions of a partner’s everyday prospective memory (PM) forgetfulness are related to the perceived cognitive support (e.g., planning, reminders) one offers the partner. Participants were 196 middle-aged and older married individuals (M = 64.51 years, Range = 40–88 years) who completed self and proxy versions of the Prospective and Retrospective Memory Questionnaire (Smith, 2000) as well as a measure of self-reported collaborative prospective memory (adapted from Reese-Melancon et al., 2018 and Harrington & Reese-Melancon, 2022). Regression analyses indicated that age and perceptions of one’s partner’s forgetfulness predicted significant variance in the amount of PM support one reports offering one’s partner. Participants who perceived greater PM forgetting in their partner reported providing more help to them, with older adults reporting providing more assistance to their partners. Additional analyses indicated biological sex and one’s own forgetfulness were significant predictors of respondents’ perceived support from their partners. Participants who reported more PM forgetting reported receiving more partner assistance with males reporting receiving more assistance than females. These findings contribute to our understanding of collaborative cognition in adulthood, especially prospective memory, an aspect of memory that is instrumental to the maintenance of independence in late life.

